# How the home learning environment contributes to children’s social–emotional competence: A moderated mediation model

**DOI:** 10.3389/fpsyg.2023.1065978

**Published:** 2023-02-14

**Authors:** Shaomei Li, Yu Tang, Yuxin Zheng

**Affiliations:** Faculty of Education, Shannxi Normal University, Xi’an, Shaanxi Province, China

**Keywords:** home learning environment, social–emotional competence, preschool children, synergistic efficacy, moderated mediation effect

## Abstract

**Introduction:**

The home learning environment is the earliest contact learning environment in early childhood development, which plays an important role in the development of children’s social-emotional competence. However, previous studies have not clarified the precise mechanisms by which the home learning environment influences children’s social-emotional competence. Therefore, the purpose of the study is to explore the relationship between the home learning environment and its intrinsic structure (i.e. structural family characteristics, parental beliefs and interests, and the educational processes) and children’s social-emotional competence, and whether gender plays a moderating role in the relationship.

**Method:**

The study randomly selected a sample of 443 children from 14 kindergartens in western China. The Home Learning Environment Questionnaire and the Chinese Inventory of Children’s Social-emotional competence scale were used to investigate the home learning environment and social-emotional competence of these children.

**Results:**

(1) Structural family characteristics and parental beliefs and interests both had a significant positive predictive effect on children’s social-emotional competence. (2) The educational processes fully mediate between structural family characteristics, parental beliefs and interests, and children’s social-emotional competence. (3) Gender moderated the effect of the home learning environment on children’s social-emotional competence. Gender moderates not only the indirect effects between parental beliefs and interests and children’s social-emotional competence, but also the indirect effects between structural family characteristics and children’s social-emotional competence. At the same time, gender also moderated the direct effects between parental beliefs and interests and children’s social-emotional competence.

**Discussion:**

The results emphasize the crucial role of the home learning environment in the development of children’s early social-emotional competence. Therefore, parents should pay attention to the home learning environment and improve their ability to create a home learning environment that promotes the positive development of children’s social-emotional competence.

## 1. Introduction

Social–emotional competence is a core competency for children’s adaptation and social development in complex situations and plays an important role in children’s overall development (Osher et al., 2016; Abrahams et al., 2019). In recent years, there has been an increased interest in identifying factors that may be related to growth in this set of skills (Wolf and McCoy, 2019), particularly during the preschool period when rapid development in social–emotional competence is most evident (McLeod et al., 2017; Murano et al., 2020). The home learning environment, as an effective combination of activities and resources at home, is a major factor in the development of children’s social–emotional competence (Domitrovich et al., 2017; Lehrl et al., 2020). Previous studies have shown that factors such as socioeconomic status, family functions, family activities, and parenting styles influence the development of young children’s social–emotional competence (Liu J. et al., 2020; Schapira and Aram, 2020; Hooper et al., 2022). However, the home learning environment is greater than just the sum of its component environmental elements. It is a multidimensional, interactive and cyclical system. Therefore, in the study, we explored the synergistic effects and developmental mechanisms of the dynamic system of the home learning environment on children’s socioemotional development.

### 1.1. Social–emotional competence

Emotional intelligence theory suggests that emotions influence the direction of attention, the processes of memory, and problem-solving (Bucich and MacCann, 2019). Social–emotional competence, based on theories such as emotional intelligence, is the characteristic and behavior of individuals who can establish and maintain positive relationships with others, fulfill the demands of the social environment, and achieve desired goals in groups. It includes cognitive control, emotional expressivity, empathy and prosocial behaviors, and emotion regulation, which are important aspects of non-cognitive abilities (Józsa and Barrett, 2018; Kwong et al., 2018). Among these, cognitive control refers to the children’s ability to respond according to the goal or task, to preserve the goal when distractions are encountered, and to inhibit habitual responses or impulsive behavior (Nigg, 2017; Frick et al., 2021). Emotional expressivity is the ability to make a distinction between internal feelings and external emotional expressions without violating the rules of emotional expression (Hajal et al., 2020). Empathy and prosocial behaviors refer to children’s ability to understand the emotions and intentions of others (Silke et al., 2018). Emotional regulation is the ability of children to induce, inhibit and maintain different emotional states (Roth et al., 2019). In general, these emotional and social skills do not operate in isolation. For example, children who are more able to regulate their own emotions, understand the perspectives of others, and express their emotions in socially acceptable ways are also more likely to maintain relationships and manage conflict.

In preschool, children’s imaginative thinking develops rapidly, abstract thinking is gradually developed, and it is a critical period for the development of social–emotional competence (Blewitt et al., 2021). Social–emotional competence predicts a variety of developmental outcomes, including academic achievement, psychological development, and interpersonal relationships (Panayiotou et al., 2019; Herrera et al., 2020; MacCann et al., 2020). In the academic achievement section, children’s learning occurs through hands-on inquiry and interaction with the external environment, and its effectiveness depends not only on the child’s intellectual level, but non-intellectual factors also play an important role (Zhu et al., 2022). Social–emotional competencies among non-cognitive skills can promote the development of good learning quality in children, influence the development of learning qualities, and improve academic performance (Moore et al., 2015; Kim and Shin, 2021). Specifically, children with social–emotional disorders perform worse in school and have lower socioeconomic status in adulthood (von Stumm and Plomin, 2021). However, when children have social–emotional competencies, they can increase motivation, increase engagement in learning, and reduce anxiety to be ready for school (Turnbull et al., 2022). Thus, it can be argued to some extent that social–emotional competence is a key aspect of school success (Domitrovich et al., 2017). In terms of psychological development, social–emotional competencies enable children to increase self-awareness, gain a fuller and clearer understanding of the connection between the external environment and self-development, motivate children to communicate with others, relate, resolve conflicts and achieve goals and lay the foundation for future success (Poulou et al., 2018; Ahmed et al., 2020). Therefore, social–emotional competence can, to a certain extent, effectively prevent and solve the psychological problems that arise during the growth of young children and help to form good personality qualities and a sound character (Jennings et al., 2019). In terms of interpersonal interactions, children with social–emotional competence are more likely to know what emotions are and how to use and manage them, understand emotional values and consequences of behavior, and express emotions rationally (Lam et al., 2022). Thus, social–emotional competence enables young children to establish and maintain good interpersonal relationships, deal effectively with a variety of interpersonal problems, and better understand the nature of social interaction (Schonert-Reichl, 2019).

Children’s social–emotional competence is affected by the interaction of multiple environments, including the school environment, home environment, and social environment (Wu et al., 2018). What’s more, the family is not only the initial environment to which children are exposed since birth, it also is an important field that influences children’s social–emotional development and is the basic unit of interaction with the external environment (Cuartas, 2022). Therefore, providing children with a stimulating home environment maximizes their opportunities to develop social–emotional skills (Ng and Bull, 2018).

Previous research has shown that elements of the home environment are independent predictors of children’s social–emotional competence development. A study of family structure found that children from two-parent families scored higher in social–emotional development compared to children from single-parent families (Wang et al., 2019). Children from low socioeconomic status families were more likely to have problems such as isolation, low self-esteem, and lack of social communication skills (Spinelli et al., 2021). Meanwhile, some researchers have looked at more microscopic factors such as family possessions and family book collections. All of these factors were found to promote the development of children’s collaborative skills, which in turn promoted children’s social–emotional competence (Tang et al., 2021). In addition to exploring the above factors that cannot be changed in the short term, more variable factors such as family climate and parenting style are also variables that have been explored more. It has been shown that poor family interpersonal climate, especially frequent spousal conflicts, may cause young children to develop negative emotions. This makes it difficult for young children to deal appropriately with their peers (Gao et al., 2019), which in turn has a negative effect on their social–emotional development. In addition, Taleb’s (2013) study found that children with positive parenting styles had better social adjustment and thus promoted their social–emotional development (Taleb, 2013). Haslam et al.’s (2020) study also confirmed this view (Haslam et al., 2020).

Studies have also examined the joint effects of different variables in the home environment on children’s social–emotional competence development. Some studies have focused on the relationship between parental variables on children’s social–emotional development, such as parenting style and parenting expectations, which were found to be related to children’s emotion regulation ability. This in turn influences the development of children’s social–emotional competence (Liu et al., 2021). In addition, scholars have found that parenting style not only has a significant positive direct effect on children’s social–emotional development through in-depth research. It can also have indirect effects through the parent–child relationship (Li and Zhang, 2022). Similar influential effects are also present in other variables in the home environment. For example, Zhang et al. (2022) and others showed that parental involvement also has a mediating role in the process of family economic status affecting children’s social–emotional competence (Liu J. et al., 2020).

A review of research on the influence of family environment on children’s social–emotional competence reveals that previous studies have focused on the influence of different variables in the home environment on children’s social–emotional competence, though. However, it only rests on the simple summation of single variables or multiple variables. It has not been taken into account that the family is a complex system that is not just a combination of parts, but an organic and interactive whole. Based on this view, we believe that the study should take a more macroscopic perspective and delve into the interactions among different variables in the home environment and how to combine these variables organically, focusing on the synergistic effects of the dynamic combination of family variables on children’s social–emotional competence development.

### 1.2. The role of the home learning environment in children’s social–emotional competence

The home learning environment, as an effective combination of educational resources and activities, is an important process variable that promotes children’s development and has a significant impact on early childhood development (Nguyen et al., 2018). Compared with the relatively static nature of the home environment, the influence of the home learning environment on children’s social–emotional competence has unique interactive, dynamic and systematic characteristics. First, interactivity refers to a pattern of interaction between parents and children through daily communication and exchange of information, opinions, emotions and attitudes. In this process, children gradually acquire the ways and means to interact with others, form the cognition of social rules, and realize the early development of social–emotional ability (Wu et al., 2018). Second, dynamism refers to the effective combination of educational resources such as family possessions and cultural background with educational activities, dynamically adjusting parenting style, updating educational content and constantly sending positive signals to children. Children can gain a greater sense of security and importance, and gradually develop a high level of self-esteem and self-efficacy. In turn, they are more willing to interact with people other than their parents and promote the development of their social–emotional skills. Third, systemic refers to the fact that the higher the parental educational expectations for their children’s social–emotional competence development in the home learning environment, the more importance they place on the children’s related development. Parents will become more frequently involved in their children’s education and influence the development of their children’s social–emotional competence in various ways. At the same time, children are able to provide more information to their parents in the process in order to improve the parenting style and content, creating a feedback loop system. Therefore, research on the home learning environment can help to understand more deeply the interaction between parents and children in the family, explore the dynamic influence of different elements in the family on social–emotional competence, and ultimately form a feedback loop system of family and children’s social–emotional competence development (Tamis-LeMonda et al., 2019).

To better explore the internal elements of the home learning environment, the conceptualization of the home learning environment in the present study is situated within the bio-ecological model of human development which delineates distal and proximal processes to affect the child’s development (Domoff et al., 2020). Based on the bioecological model of human development, we hypothesize that distal processes indirectly influence children’s development and are mediated by proximal processes, which are the theoretical basis for the conceptualization model proposed in this study (Xie et al., 2021). Specifically, distal processes refer to structural family characteristics such as home language education, income, and occupational status, and also include personal factors such as parents’ educational beliefs and interests (Junge et al., 2021). In addition, parents’ general educational values, opinions about specific areas of children’s development, and expectations for children’s future academic careers are all components of parents’ educational beliefs (Ren et al., 2021). The proximal processes refer to the interaction between parents and children in the educational processes, which is the “engine” of development. It reports a strategic position in the home learning environment and can compensate to some extent for the negative factors in the family, esspecially the adverse effects of factors that cannot be changed in the short term, such as socioeconomic status and preschool experience in early childhood development, which is particularly important to child development (Stahl et al., 2018). Therefore, based on previous studies, the home learning environment was conceptualized into three dimensions: structural family characteristics, parental beliefs and interests, and educational processes. Among these, the educational processes were placed at the core of the home learning environment (Kluczniok et al., 2013).

The educational process refers to the combination of proximal parenting behaviors that parents provide for their children. It often encompasses two aspects. On the one hand, it refers to parental behaviors that support the overall social–emotional interactions within the family. For example, verbal support, material support, psychological support etc. (Di Giorgio et al., 2021). On the other hand, it refers to learning activities that parents provide for children in the home. For instance, activities such as playing with blocks, reading, singing, and learning about knowledge (Lee et al., 2021). Previous researches have demonstrated that the educational processes have a positive impact on children’s social–emotional development (Yang et al., 2019). Children are not born with social–emotional competence, but acquire it through interactions with caregivers after birth (Housman, 2017). In general, children’s development benefits from high-quality learning materials and a high frequency of stimulating interactions. Through interactions with caregivers, children progressively make many of the connections in their brains associated with the development of social behaviors (Tan et al., 2020). However, if caregivers do not provide appropriate, consistent, and complementary parenting behaviors, the children’s social–emotional competence development will be disrupted (Shi et al., 2021). The results of related studies (Mistry et al., 2008) confirm the idea that the activities parents do at home with their children are more important for the development of children’s social skills than who they are.

Structural family characteristics refer to stable and persistent features in the family context (Horning et al., 2022). Previous studies have mostly characterized the family in terms of its socioeconomic background, cultural background, family composition, parents’ education level, occupation, and several other factors components (Hummel et al., 2022). Research has shown that structural family characteristics significantly influence children’s social–emotional competence. From the social capital theory perspective, human competencies are formed through human investment, and the human investment that a family can afford is largely dependent on the economic capital as well as the human capital of that family (Gannon and Roberts, 2020). Families with high economic levels can provide both adequate resources and a safe environment for child development (Liu Q. et al., 2020). In contrast, low-income families are significantly more likely to have children with social–emotional and behavioral problems due to the greater stresses of life (Pace et al., 2019). Subsequent studies also validate this finding that children from poor families have significant underdevelopment of social–emotional competence before they enter school compared to children from high-income families and that the gap in children’s emotional competence due to family income widens progressively with age (Bender et al., 2022). Parental education level is also a component of structural family characteristics, and studies have demonstrated that parental education level significantly predicts children’s social competence, and the predictive effect is somewhat stable (Albanese et al., 2019).

Parental beliefs and interests include beliefs about self and interests in children development (Kim et al., 2013). Specifically, the former refers primarily to parents’ perceived responsibility for themselves, including how they should be involved in their children’s education, and school activities, or how they can help their children with their schoolwork. Previous studies have focused on parental beliefs about the ways in which they can engage in home-school cooperation and home-education activities (Tett and Macleod, 2020). The latter mainly refers to parental interests in the fact that they can effectively influence their children’s school success, such as feeling empowered in helping their children learn, etc. (Muenks et al., 2018). Existing research has more often explored parental interests in their children’s academic achievement, school readiness, adaptability, and social competence (Puccioni, 2018). Parental beliefs and interests can predict their children’s later cognitive, language, and social–emotional outcomes (Kinsler and Pavan, 2021; List et al., 2021; Ronderos et al., 2022). Self-efficacy theory suggests that parental beliefs are essential to child development (Bonanati and Buhl, 2022; Kong and Yasmin, 2022). That means the progress of children’s social skills and social–emotional competencies can be promoted by increasing parents’ beliefs and interests in their children’s education (Fung, 2022). Parents with high educational beliefs not only have higher educational expectations for their children but also have higher expectations for their education (Dong et al., 2020; Lau and Lee, 2021). This often results in greater parental involvement in the children’s educational activities and sends positive signals to the child, thereby enhancing the children’s social–emotional competence (Ren et al., 2019). However, there are few studies related to parental beliefs and children’s social–emotional competence, and the mechanism of the interaction between the two needs to be further clarified. Therefore, exploring the relationship between parental beliefs and children’s social–emotional competence can also appropriately complement research in related fields.

Therefore, both structural family characteristics, parental beliefs and interests, and educational processes are deemed to be to the development of preschool children’s social–emotional competence. However, the present research on the influence of the home learning environment on children’s social–emotional competence is scarce, and most studies have investigated only single elements of the home learning environment, such as the impact of structural family characteristics and children’s social–emotional competence. Little is known about the influence of parental beliefs and interests, educational processes, and other factors. In particular, it has not been clarified how the three dimensions within the home learning environment interact with each other and influence children’s social–emotional competence development. Therefore, the purpose of this study was to investigate how the home learning environment is related to children’s social–emotional competence at ages 3–6 by investigating different elements of structural family characteristics, parental beliefs and interests, and educational processes.

### 1.3. Gender as a moderator

In addition, a substantial body of evidence suggests that there are gender differences in the development of social–emotional competence in Chinese children. In terms of the different aspects of children’s social–emotional competence development, Gao et al. divided children’s problem behaviors into two parts: internalizing and externalizing problems. And they found that among Chinese preschoolers, boys had significantly more externalizing problems than girls. This may lead to better development of social–emotional skills in girls than in boys (Gao et al., 2018). Some studies have also researched children’s social–emotional competence by investigating their development in the areas of social compliance, nonaggression, empathy, and helpfulness. It was found that girls in the preschool years performed significantly better than boys in all these areas. Following this line of thought, scholars have divided social–emotional competence into different dimensions as a way to explore whether there are gender differences in its development. Huang found that boys and girls differ in social behavior, emotional regulation, and emotional control (Huang et al., 2021). Liu et al. have further explored that boys performed better than girls on the emotion regulation dimension, especially on the resilience, which was significantly higher than girls (Liu et al., 2021). In addition, some researchers categorized the social–emotional competencies into five competencies: self-awareness, self-management, social awareness, interaction skills, and making decisions. It was also found that Chinese elementary school girls had significantly higher mean scores on all five social–emotional competencies than boys. The different test also showed that the difference between both on the five major competencies reached the level of significance (Chen, 2021). Further evidence that there are gender differences in the development of children’s social–emotional competence. And in terms of the time point of children’s social–emotional competence development, girls may develop social–emotional competence earlier than boys. This is evidenced by the fact that preschool boys are more withdrawn, more aggressive, and less social–emotionally competent than girls in China (Chen and Jiang, 2002). It is worth mentioning that a different conclusion was reached by Tang. The study explored the current status of social–emotional competence in children of different ages and genders. It was concluded that there were significant differences in the collaborative abilities of children by gender, with girls being significantly higher than boys in the 10-year-old group and boys being higher than girls in the 15-year-old group (Tang et al., 2021).

However, due to traditional views and societal gender stereotypes, Chinese parents often exhibit different educational concepts and approaches when dealing with children of different genders. In parenting expectations, influenced by gender role theory, parents tend to view boys as independent, strong. In contrast, the girl is dependent, soft, and emotional. As a result, parents have different career expectations for their children of different genders. Parents tend to believe that boys should be in adventurous, exploratory, and relatively independent jobs. Girls, on the other hand, should be in safer, more stable occupations that involve more interaction with others. As a result, parents approach the education of their children differently by gender. Parents tend to adopt a more authoritarian parenting style when dealing with boys, using a command style of communication to communicate. In contrast, the process with girls involves more emotional communication and uses more positive and negative emotional expressions (Xu et al., 2022). As a result, girls tend to experience parental warmth and care more readily and are encouraged to show empathy and care for others. In contrast, along with more parental control, boys may perceive pro-social behavior as an externally imposed obligation that affects their development of pro-social behavior (Zhao et al., 2023). At the same time, parents may engage in different types of educational activities with their children of different genders. This is evidenced by the fact that boys tend to go outdoors with their parents and engage in some exploratory activities. Even at home, they tend to engage in activities that can be done independently, such as puzzles, building blocks, and so on. For girls, the educational activities usually take place at home, where parents, especially mothers, often guide girls to participate in more interactive activities, such as role-playing games. Such different educational behaviors and approaches tend to make girls more likely than boys to have opportunities to interact with others, to acquire social interactions, to exhibit more pro-social behaviors, and to be more willing and adept at interacting with others. This may ultimately affect the development of children’s social–emotional competence.

And factors such as educational processes in the family, parental beliefs and parenting styles are also part of the home learning environment. Therefore, we hypothesize that there are gender differences in the way the home learning environment influences children’s social–emotional competence in the Chinese context. However, in what ways such differences manifest themselves, we do not explore that. This issue is worth further understanding and investigation. In particular, many social-psychology trait differences are actually implied behind the gender factor (Morgenroth and Ryan, 2018). In this sense, the present study realizes that gender is more suitable as a moderating variable.

### 1.4. The present study

Therefore, the purpose of this study was to investigate how the home learning environment is associated with children’s social–emotional competence and whether gender plays a moderating role in it by investigating different elements such as family structural characteristics, parental beliefs and interests, and the educational processes.

Taken together, children’s social–emotional competence is malleable and there is considerable evidence of the important role which the home learning environment plays in the development of children’s social–emotional competence (Brophy-Herb et al., 2019). However, the current research has the following flaws:

First, there are relatively few studies that have examined the current state of development of social–emotional competence in preschool children, especially in the Eastern cultural context, and little is known about the development of social–emotional competence in children aged 3–6 years. Second, although many researchers have interpreted the multidimensional connotation of home learning environment based on national cultural backgrounds and their value orientation, a general definition or a common operational definition of home learning environment is still missing, especially when there are cases of confusion and misuse of the connotation of the home environment and home learning environment. Therefore, it is necessary to systematically analyze the conceptual framework of the home learning environment to provide a theoretical basis for the formation of the home learning environment and child development mechanism. Third, most of the existing studies have examined only the effects of home learning activities related to the home learning environment or a particular home learning environment factor on children’s development. To date, no studies have simultaneously considered the interaction of different factors in the home learning environment and their influence on children’s social–emotional competence. More specifically, are there any relationships between the educational processes and structural family characteristics, as well as parental beliefs and interests in the home learning environment, relating to the educational processes? And the extent to which the educational processes mediate the effects of structural family characteristics and parental beliefs and interests on children’s social–emotional competence has yet to be clarified. Fourth, most studies have examined gender differences in children’s social–emotional competence in elementary school and beyond, but it does not know whether gender differences are significant in the preschool years. It is not known whether the influence of home learning environment and its internal factors on preschool children’s social–emotional ability is regulated by gender.

**Figure 1:**
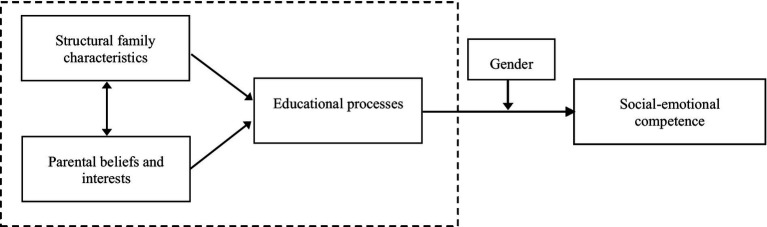
A hypothesized moderated mediation model (Adapted from Kluczniok et al., 2013).

Therefore, in this study, we proposed a hypothetical model as shown in Figure 1, proposing to explore the interactions of elements within the home learning environment and whether the educational process (mediating variable) among them provides a mechanism through which structural family characteristics (independent variable 1) and parental beliefs and interests (independent variable 2) are indirectly related to children’s social–emotional competence (dependent variable) at the age of 3–6 years. Also, exploring the effect between the home learning environment and children’s social–emotional competence with gender as a moderating variable is the focus of the research.

We proposed the following hypotheses.

*H1*: Structural family characteristics and parental beliefs and interests would be positively related to children’s social-emotional competence.

*H2*: The educational processes would mediate the link between structural family characteristics, parental beliefs and interests, and children’s social-emotional competence.

*H3*: Gender would moderate the influence of the home learning environment on children’s social-emotional competence. Specifically, gender would moderate the direct effects of parental beliefs and interests on children’s social-emotional competence and also moderate the indirect effects between the two through the educational processes (H3a). In addition, gender would moderate the direct effects of structural family characteristics on children's social-emotional competence and likewise moderate the indirect effects between the two through the educational processes (H3b).

## 2. Methods

### 2.1. Participants

Participants included 443 preschool children and one of their parents recruited from a random sample of 14 kindergartens in western China to take part in the survey. The sample was comprised of 173 boys and 270 girls. Children ranged in age from 37 to 87 months (*M* = 61.38, SD = 3.86). In China, kindergartens are typically 3-year programs for children aged 3–6 years. As with most kindergartens in China, all kindergartens in this study offered full-day programs, and children were located in school for approximately 8 h per day.

### 2.2. Measures

#### 2.2.1. Social–emotional competence

Children’s social–emotional competence was accessed using the Chinese Inventory of Children’s Social–emotional competence (CICESC) developed by Li et al. (2020). The scale Cronbach’s alpha coefficient was 0.92, with good reliability and validity. The scale was in the form of a kindergarten teacher report and included four dimensions of cognitive control (six items), emotional expressivity (seven items), empathy and prosocial behaviors (11 items), and emotion regulation (six items), with a total of 30 questions. Teachers rated the children’s performance on the relevant dimensions from 1 (strongly disagree) to 5 (strongly agree), and the ratings were averaged, with higher scores indicating higher levels of children’s social–emotional competence.

##### Cognitive control

The cognitive control dimension of the scale assessed two abilities of young children: the ability to maintain information in short-term memory to complete a task, and the ability to remain focused without external influences. This component consisted of five working memory items and one inhibitory control item. The Cronbach’s alpha coefficient was 0.88.

##### Emotional expressivity

Emotional expressivity assessed children’s ability to make a point without violating the principles of emotional venting. This section consisted of four items on emotional expressivity, two items on relationship building, and one item on inhibitory control. The Cronbach’s alpha coefficient was 0.87.

##### Empathy and prosocial behaviors

This dimension rated two main areas of development: children’s abilities to understand the emotions and intentions of others, and their willingness to comfort and help others. These consisted of four emotion-understanding items, five relationship building items, and two conflict management items. The Cronbach’s alpha coefficient was 0.90.

##### Emotion regulation

This dimension assessed two parts: children’s abilities to regulate their negative emotions and adapt to changes without showing negative emotions. These consisted of four emotion regulation items and two cognitive flexibility items. The Cronbach’s alpha coefficient was 0.83.

#### 2.2.2. Home learning environment

Children’s home learning environment was accessed using the Home Learning Environment Questionnaire developed by Kluczniok et al. (2013). And the study revised it according to the actual characteristics of Chinese children’s home learning environment. The scale was in the form of a parent report and included three dimensions of structural family characteristics (five items), parental beliefs and interests (eight items), and educational processes (17 items), with a total of 30 items. The reliability and validity of the questionnaire were obtained after pretesting with expert opinion counting, indicating good reliability (Cronbach’s alpha = 0.824).

##### Structural family characteristics

These mainly refer to stable and long-lasting characteristics of the family background, including both parental education and income. Parental education includes items such as parents’ level of education and occupation. The scale consists of four items, numbered 1–4. The Cronbach’s alpha coefficient was 0.79. Income is disposable household income *per capita* (socioeconomic background), including item number 5.

##### Parental beliefs and interests

Parental beliefs and interests in the questionnaire refer to educational beliefs and interests that help stimulate children’s learning and include two components. One is parental beliefs about themselves, which mainly assesses the degree to which parents value different educational activities. For example, educational activities such as motor skills, language skills, social skills, creative skills, and personality development. The scale consists of five items, numbered 6–10. The Cronbach’s alpha coefficient was 0.90. The second is the parental interest in child development, which focuses on assessing their own level of competency in helping their children with educational activities. For example, performing educational activities such as cognitive skills, basic skills, and school readiness. The scale consists of three items, numbered 11–13. The parents were asked to rank, on a five-point scale (1 = not important to 5 = very important), how important different domains of stimulation are from their perspective. The Cronbach’s alpha coefficient was 0.82.

##### Educational processes

The educational processes consist of two main components: family support and home learning activities. Family support includes material support and non-material support and includes six items, numbered14-19. Examples include: verbal support, material support, psychological support, etc. The Cronbach’s alpha coefficient was 0.83. Home learning activities measured the frequency of activities such as reading and reading, singing, playing with toys, outdoor activities, etc. (1 = never; 5 = always). The scale consists of 11 items, numbered 20–30. The Cronbach’s alpha coefficient was 0.87.

#### 2.2.3. Procedure

All of the procedures performed in this study were by the 1964 Helsinki declaration and its later amendments and with the APA ethical standards. For the selection of the study object, the authors contacted public kindergartens in western China, mainly including Shaanxi, Guizhou, and Sichuan provinces, through their personal networks (i.e., based on convenience samples). These provinces are located in the northwest and southwest of mainland China, with a relatively large population base and a more diverse demographic composition. This allows the public kindergartens in these regions to recruit students from different provinces and ethnic groups. And this also renders our research to have an opportunity to recruit participants from different regions of China, which may lessen the potential influence of intercultural differences on the outcome of the study and make the findings more representative of the overall population of Chinese preschoolers. After a long process of negotiation, directors of 14 public kindergartens in the western region finally agreed to collaborate with us in this study. Therefore, from June 2022 to September 2022, the research team conducted a questionnaire campaign for 3 months. Specifically, a random sample of 445 children and one of their parents from 14 public kindergartens in western China was used for this study. Among them, 443 questionnaires were valid, with an effective rate of 99.6%. The researcher invited teachers and parents to participate in the study. Consent forms were obtained from parents before administering child assessments and delivering questionnaires to families’ homes. After obtaining informed consent, parents of preschool children completed the questionnaires about the home learning environment. And 99% of the questionnaires were parent-reported. And then, teachers were paired to complete the CICESC for the sampled children based on the completed parent questionnaires. That is, the children’s behavior was rated on a scale of 1–5 based on their performance in school.

#### 2.2.4. Statistical analysis

**Figure 2:**
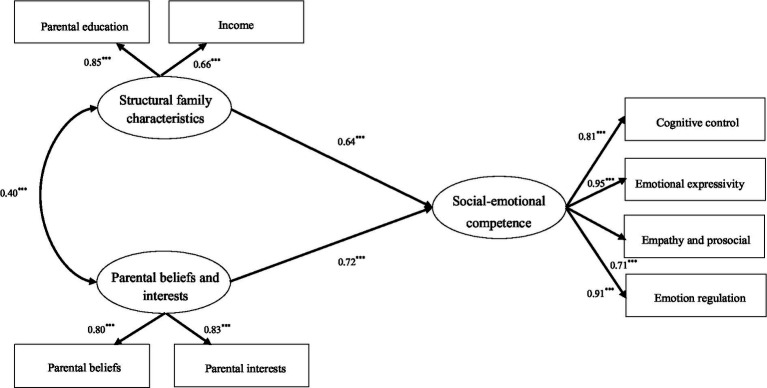
The standardized path coefficients describe the direct predictive effects of structural family characteristics, parental beliefs and interests on children’s social–emotional competence. ***p < 0.001.

Firstly, we employed SPSS25 software to analyze descriptive statistical and correlations. Secondly, we used AMOS24 software to construct Structural Equation Model to investigate the mediating role of the educational process between parental beliefs and interests, structural family characteristics, and children’s social–emotional competence. The research estimated the fit of the model and tested hypotheses on the causality model of the model. Specifically, we developed a path model as shown in Figure 2. The model was specified in such a way that structural family characteristics and parental educational beliefs and interests were the independent variables, social–emotional competence was the dependent variable, and the educational processes were the mediating variable. The study examined the four latent variables together in the same model to the specific pathways by which each element of the home learning environment acts to influence children’s social–emotional competence. In addition, the independent variables are predictors of the mediating and dependent variables. The mediating variable is equally a predictor of the dependent variable. The direct effect is the path from the independent variable to the dependent variable. The indirect effect is the result of the path from the independent variable to the mediating variable and the path from the mediating variable to the dependent variable. Finally, we employed Model 59 of the PROCESS to conduct moderated mediation analysis so as to decide whether the indirect path was moderated by gender.

## 3. Results

### 3.1. Descriptive data and correlations

Descriptive statistics and correlations between the dimensions of the home learning environment and social–emotional competence are presented in Table 1. Specifically, social–emotional competence showed positive correlations with parental education, income, parental beliefs, parental interests, family support, and home learning activities in the home learning environment. In addition, age was found to be significantly and positively correlated with parental beliefs, parental interests, and children’s social–emotional competence. This suggests that the stronger the parental beliefs and parental interests of parents of older children, the stronger their social–emotional competence. Also, family support and home learning activities were negatively correlated with the number of children in the family. This indicates that the higher the number of children in the family, the lower the quality of family support and home learning activities, and the lower the social–emotional competence of children. Furthermore, in the correlation analysis, the category variable gender was not statistically significantly correlated with any of the other variables. That is, there were no significant gender differences in the social–emotional competence of the children in this study. This may be due to the fact that the initial development of social–emotional competence in children aged 3–6 years did not show significant differences. However, gender can still be used as a moderating variable for further analysis of the moderating effect.

**Table 1:** Descriptive statistics and correlations among variables.

	1	2	3	4	5	6	7	8	9	10
1. Age	–									
2. Number of children	0.14	–								
3. Gender	0.01	−0.04	–							
4. Parental education	0.07	−0.20***	0.04	–						
5. Income	0.05	−0.29***	0.07	0.39***	–					
6. Parental beliefs	0.20***	−0.03	0.02	0.30***	0.14***	–				
7. Parental interests	0.20***	−0.04	0.01	0.32***	0.16***	0.82***	–			
8. Family support	0.06	−0.32***	0.04	0.56***	0.39***	0.47***	0.43***	–		
9. Home learning activities	0.04	−0.41***	0.03	0.33***	0.27***	0.34***	0.39***	0.64***	–	
10. Children’s social competence	0.13***	−0.20***	0.03	0.35***	0.22***	0.33***	0.32***	0.71***	0.47***	–
*M*	2.36	1.67	1.61	3.55	2.89	4.29	4.29	4.29	4.28	3.44
SD	0.71	0.52	0.49	0.58	0.73	0.76	0.70	0.50	0.63	0.85

*N* = 443. *p < 0.1, **p < 0.01, ***p < 0.001.

### 3.2. Mediation analyses

The latent variable structural equation model developed in this study consists of two parts, the measurement model and the structural model. Among them, a good fit of the measurement model is a prerequisite for a satisfactory fit of the comprehensive model. Therefore, this study tested the measurement model first, and then the structural model. According to the mediation effect test procedure, the direct effect test of structural family characteristics and parental educational beliefs and interests on the prediction of children’s social–emotional competence was done first, followed by the structural model mediation effect test of adding the educational process. Previous research has found that age and number of children in the family all contribute to significant differences in children’s social–emotional competence. Therefore, this study controlled for the above demographic variables.

**Table 2:** Standardized estimates for direct and indirect effects of variables.

Path	Est.	SE	95% CI	
			Lower	Upper
Direct effects				
SFC → SEC	0.64***	0.07	0.02	0.03
PBI → SEC	0.72***	0.10	0.02	0.09
SFC → EP	0.66***	0.18	0.55	0.80
PBI → EP	0.64***	0.09	0.10	0.35
EP → SEC	0.88***	0.18	0.09	0.23
Indirect effects				
SFC → EP → SEC	0.58***(a*b)	0.05	0.42	0.99
PBI → EP → SEC	0.56***(a*b)	0.09	0.09	0.33

*N* = 443. SFC, Structural family characteristics; PBI, Parental beliefs and interests; EP, Educational processes; SEC, social–emotional competence; Est., standardized estimates; CI, confidence interval, ***p < 0.001.

#### 3.2.1. A test of the direct predictive effects of structural family characteristics, parental beliefs and interests on children’s social–emotional competence

A model test of the direct effects of structural family characteristics and parental educational beliefs and interests on children’s social–emotional competence is illustrated in Figure 2. The results revealed a good model fit (*χ*^2^ = 97.210, *df* = 27, *χ*^2^/*df* = 3.60, *p* < 0.001, RMSEA = 0.055, GFI = 0.908, IFI = 0.912, NFI = 0.904, CFI = 0.911, PNGI = 0.591, PGFI = 0.582). It is also evident from the data in Table 3 that both structural family characteristics (*β* = 0.64, *SE* = 0.07, *t* = 7.41, *p* < 0.001) and parental beliefs and interests (*β* = 0.72, *SE* = 0.10, *t* = 8.53, *p* < 0.001) had a significant positive predictive effect on children’s social–emotional competence, and Hypothesis 1 was supported.

**Table 3:** Tests of the moderated mediation effect A.

	EP			SEC		
	*β*	SE	95% CI	*β*	SE	95% CI
Age	−0.01	0.03	−0.07, 0.05	0.22	0.05***	0.05, 0.32
Number of children	−0.44	0.05***	−0.54, −0.35	0.10	0.07	−0.03, 0.24
Gender	0.01	0.05	−0.08, 0.10	0.03	0.07	−0.10, 0.17
PBI	0.30	0.04***	0.23, 0.37	0.17	0.05***	0.08, 0.27
EP				0.81	0.07***	0.68, 0.95
PBI * Gender	−0.01	0.07	−0.14, 0.13	0.26	0.10**	0.08, 0.46
EP * Gender				0.44	0.13***	0.18, 0.68
*R* ^2^	0.57			0.65		
*F*	64.30***			63.79***		

*N* = 443. SFC, Structural family characteristics; PBI, Parental beliefs and interests; EP, Educational processes; SEC, social–emotional competence; Est., standardized estimates; CI, confidence interval; *p < 0.1; **p < 0.01; ***p < 0.001.

#### 3.2.2. A test of the indirect predictive effects of structural family characteristics, parental educational beliefs and interests on children’s social–emotional competence: An analysis of mediating effects of educational processes

**Figure 3:**
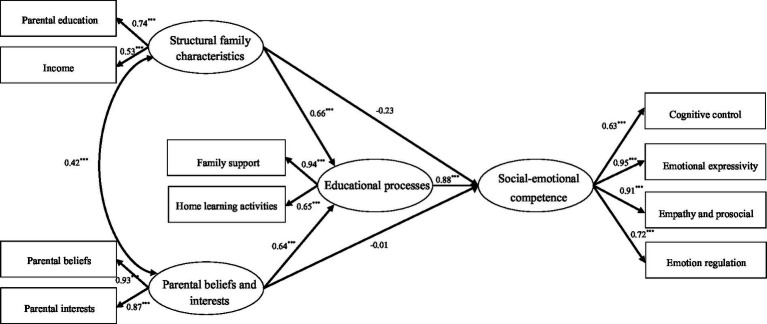
The standardized path coefficients describe the mediating role of the educational process between structural family characteristics, parental beliefs and interests and the children’s social–emotional competence. ***p < 0.001.

The model examined the effects of internal elements of the home learning environment on children’s social–emotional competence using structural family characteristics and parents’ educational beliefs and interests as independent variables, educational processes as mediating variables, and children’s social–emotional competence as dependent variables, as shown in Figure 3. The results of the study showed that the comprehensive model fit index was good (*χ*^2^ = 108.029, *df* = 29, *χ*^2^/*df* = 3.73, *p* < 0.001, RMSEA = 0.048, GFI = 0.982, AGFI = 0.938, IFI = 0.912, NFI = 0.903, TLI = 0.956, PNGI = 0.591, PGFI = 0.582). Therefore, we further assessed the mediating effects.

Further analysis of the model paths revealed a significant positive predictive effect of educational processes on children’s social–emotional competence (*β* = 0.88, *SE* = 0.18, *t* = 6.22, *p* < 0.001). The path coefficients of structural family characteristics (*β* = 0.66, *SE* = 0.18, *t* = 7.41, *p* < 0.001), parental educational beliefs and interests (*β* = 0.64, *SE* = 0.09, *t* = 8.49, *p* < 0.001) were significant for the educational process. However, the path coefficients of both structural family characteristics (*β =* −0.23, *SE* = 0.05, *p* > 0.05) and parental educational beliefs and interests (*β* = −0.01, *SE* = 0.09, *p* > 0.05) on children’s social–emotional competence became insignificant. This suggests that the predictive effects of structural family characteristics and parental beliefs and interests on children’s social–emotional competence are mediated exclusively by the educational processes.

The significance of the structural model mediation effect was verified by using the Bootstrap method of Bias-Corrected Bootstrap with a randomly repeated sample of 5,000 for estimation. As seen in Table 2, the 95% confidence interval for each indirect effect estimate did not include 0 and was statistically significant. Thus, the mediating effect of the educational processes in parental educational beliefs and interests on children’s social–emotional competence was significant. Hypothesis 2 was supported.

### 3.3. Moderating mediation analyses

We employed Model 59 of PROCESS (Hayes et al., 2017) to investigate whether the mediation effect of the educational process was moderated by gender. As seen in Table 3, after controlling covariates (age and the number of children), the educational process was significantly predicted by parental briefs and interests (*β* = 0.30, *p* < 0.001), but not by the interaction effect of parental briefs and interests and gender (*β* = −0.01, *p* > 0.05). The direct effect of the educational process on children’s social–emotional competence was significant (*β* = 0.81, *p* < 0.001), and there was a positive and significant moderation effect of gender between the educational process and children’s social–emotional (*β* = 0.44, *p* < 0.001). Moreover, gender was also found to moderate the direct effect of parental briefs and interests on children’s social–emotional competence (*β* = 0.26, *p* < 0.01). These observations suggested that both the direct and indirect association between parental briefs and interests and children’s social–emotional was moderated by gender. More specifically, this was a second stage moderated mediation model, which linked the educational process and children’s social–emotional. Thus, Hypothesis 3a was partially supported.

**Table 4:** Tests of the moderated mediation effect B.

	EP			SEC		
	*β*	SE	95%CI	*β*	SE	95%CI
Age	0.02	0.04	−0.05, 0.09	0.18	0.05***	0.05, 0.29
Number of children	−0.41	0.05***	−0.50, −0.31	0.14	0.07	−0.01, 0.28
Gender	0.01	0.05	−0.09, 0.11	0.03	0.07	−0.11, 0.17
SFC	0.22	0.03***	0.16, 0.29	0.02	0.05*	−0.17, −0.08
EP				0.84	0.07***	0.71, 0.97
SFC * Gender	−0.02	0.07	−0.15, 0.10	0.13	0.10	−0.07, 0.34
EP * Gender				0.51	0.13***	0.26, 0.76
*R* ^2^	0.51			0.63		
*F*	34.56***			52.38***		

*N* = 443. SFC, Structural family characteristics; PBI, Parental beliefs and interests; EP, Educational processes; SEC, social–emotional competence; Est., standardized estimates; CI, confidence interval. *p < 0.1; **p < 0.01; ***p < 0.001.

**Figure 4:**
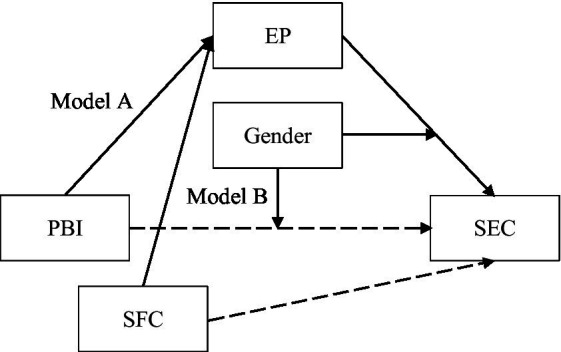
The moderated mediation model. PBI, parental beliefs and interests; SFC, structural family characteristics; EP, educational process; SEC, children’s social–emotional competence. Model A: Gender moderates PBI, EP, and SEC. Model B: Gender moderates SFC, EP, and SEC.

The same, as seen in Table 4, after controlling covariates, the educational process was significantly predicted by structural family characteristics (*β* = 0.22, *p* < 0.001), but not by the interaction effect of structural family characteristics and gender (*β* = −0.02, *p* > 0.05). Consistent with the previous studies, the direct effect of the educational process on children’s social–emotional competence was significant (*β* = 0.84, *p* < 0.001), and there was a positive and significant moderation effect of gender between the educational process and children’s social–emotional (*β* = 0.51, *p* < 0.001). Moreover, the direct impact of structural family characteristics on children’s social–emotional competence is significant (*β* = 0.02, *p* < 0.1). However, the interaction effect of structural family characteristics and gender did not predict children’s social–emotional competence (*β* = 0.13, *p* > 0.5). These results suggest that gender moderates the relationship between structural family characteristics and children’s social–emotional competence, but only in the second stage of the mediating process. Therefore, Hypothesis 3b was partially supported. The specific moderated mediation model is shown in Figure 4.

**Figure 5:**
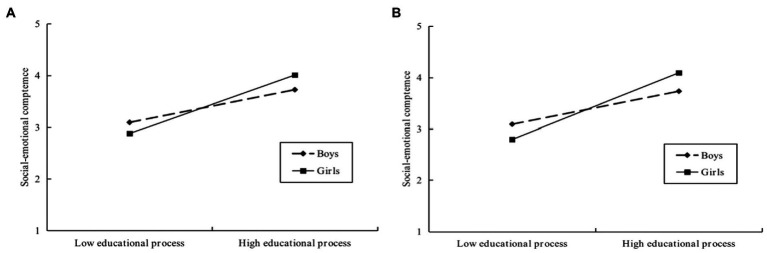
The moderating effect of gender on the relationship between educational processes and preschool children’s social–emotional competence: Simple slope, the pick-a-point approach. **(A)**: Simple slope for Model a, gender moderates PBI, EP and SEC. **(B)**: Simple slope of Model b, gender moderates SFC, EP and SEC.

**Figure 6:**
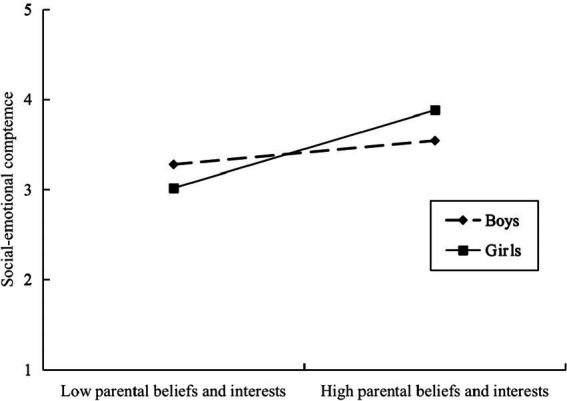
The moderating effect of children’s gender on the relationship between parental beliefs and interests and children’s social–emotional competence: Simple slope for a Model A, the pick-a-point approach.

To further validate the moderating effect, we conducted a simple slope test. As shown in Figure 5A, in Model A, the relationship between the educational processes and social–emotional competence was significant for girls (*β*_simple_ = 0.54, 95%CI = [0.35, 0.74], *p* < 0.001). However, for boys, this relationship was considerably weaker (*β*_simple_ = 0.18, 95%CI = [0.14, 0.12], *p* < 0.001). In Model B, this result also persisted. Furthermore, Figure 6 shows that the direct effect of parents’ educational beliefs and interests on girls’ social–emotional competence was also significant (*β*_simple_ = 0.57, 95%CI = [0.44, 0.70], *p* < 0.001). And, for boys, this effect was also much weaker (*β*_simple_ = 0.18, 95%CI = [0.01, 0.34], *p* < 0.001).

Analysis of conditional indirect effect analysis further illustrated that the whole indirect effect was more noticeable for girls (*β*_simple_ = 0.29, 95%CI = [0.22, 0.38], *p* < 0.001), than for boys (*β*_simple_ = 0.16, 95%CI = [0.09, 0.24], *p* < 0.001), in Model A, with a difference of 0.13, 95%CI = [0.06, 0.22], *p* < 0.001. In Model B, this result is also applicable to girls (*β*_simple_ = 0.25, 95%CI = [0.17, 0.33], *p* < 0.001) than to boys (*β*_simple_ = 0.12, 95%CI = [0.07, 0.19], *p* < 0.001), with a difference of 0.13, 95%CI = [0.07, 0.19], *p* < 0.001.

In sum, the moderated mediation model was established. The educational progress plays a mediating role in structural family characteristics, parental beliefs and interests, and children’s social–emotional competence. What’s more, gender not only moderates the educational progress and children’s social–emotional competence but also moderates the relationship between parental briefs and interests and children’s social–emotional competence.

## 4. Discussion

We investigated the relationship between the home learning environment and the social–emotional competence of children aged 3–6 by constructing a moderated mediating effect model. In summary, there were three main findings. Firstly, structural family characteristics, parental beliefs and interests were significantly correlated with preschoolers’ social–emotional competence. Thus, Hypothesis 1 was supported. Secondly, the educational processes mediated the relationship between structural family characteristics, parental beliefs and interests and preschool children’s social–emotional in which Hypothesis 2 was supported. Thirdly, gender moderated the effect of the home learning environment on children’s social–emotional competence. In model a, gender moderated not only the direct effect between parental beliefs and interests on children’s social–emotional competence, but also the second half of the pathway between the two indirect effects through the educational process. Hypothesis 3a was partially supported. In model b, gender moderated only the second half of the pathway of the mediating effect of the educational process on family structural characteristics and children’s social–emotional competence partially testing Hypothesis 3b. Generally, the findings supported the main hypotheses of this study. And it is further showed that the educational processes are the center of the home learning environment. By establishing a positive interaction between structural family characteristics and parental beliefs and interests, the development of children’s social–emotional competence can be enhanced by improving the educational processes (Sheridan et al., 2019; Cohen and Anders, 2020). The results of this study are in agreement with previous research in other areas which support the hypothesis that the internal elements of the home learning environment are interacting and have a significant impact on children’s non-cognitive abilities (Junge et al., 2021).

### 4.1. The direct impact of structural family characteristics on social–emotional competence

The results of this study are consistent with previous research findings that structural family characteristics have a significant effect on children’s social–emotional competence development. That is, social–emotional competence increases as parents’ education and household disposable income increase (Finch et al., 2018; Gadaire et al., 2021).

Firstly, in the aspect of parental education, the higher the level of parental education, the better the social–emotional competence of the children. This finding reinforces the previous findings. That means parental education significantly predicted children’s academic achievement and social competence development, and the predictive was a little stable (Boonk et al., 2018; Labella et al., 2019). Studies have shown that parents who continue additional education after birth of their children improve the quality of the home learning environment, influencing the development of children’s social emotional competence. And their children’s social–emotional competence also leads children with less educated parents (Davis-Kean et al., 2021). In addition, there has a study categorized a sample of mothers into different social class groups depending on the father’s education and occupation, from highest to lowest. The results showed that parents in higher social class groups placed a high value on their children’s development not only in the linguistic and cognitive domains, but also in the autonomy and social domains, compared to parents in lower social class groups (Eccles and Wigfield, 2020). This shows that parents of different cultures and social classes place different levels of importance to children’s social–emotional development (Im et al., 2019). These results can be made clear that parents with high levels of education may be more stimulating to children than parents with low levels of education. Combined with cultural and human capital theory, we discuss that the cultural capital of parents influences the home environment and the activities between parents and children. If parents have high levels of education, they will have a rich collection of family possessions and books, which in turn will be more apt to help and motivate children. Children will also have more access to cultural capital, which in turn helps preschoolers better identify emotions and promotes the development of children’s social–emotional competence (Jukes et al., 2021). In contrast, compared to their peers, children of parents with lower levels of education are more likely to have behavioral, emotional, and psychological problems. To some extent, this hinders the development of children’s social–emotional competencies (Schmiedeberg and Schumann, 2019).

Secondly, in the aspects of income, human capabilities are formed through human investment, and the human investment that a family can afford depends largely on the economic capital of that family from the perspective of capital theory (Jiang, 2019). The findings suggest that families with higher household disposable income provide sufficient resource for children development and more opportunities to enhance children’s social–emotional competence. Low-income families have limited access to economic resources and may be at increased risk of exposure to trauma and violence, which in part have a negative impact on children’s social–emotional competence (Homer et al., 2022). And this inequality develops in early childhood. This finding is in agreement with previous studies. Young children who grow up in poverty are at increased risk for emotional recognition, self-regulation, and social skills, which are all important factors in social–emotional competence associated with school success (Bierman and Sanders, 2021). For example, a study by Fletcher found a significant positive correlation between family income and children’s self-control and interpersonal skills, among others, and further testing found the association between family income and these skills. The association between income and these skills was found to be robust, i.e., children from higher-income families have better development of social–emotional competence (Fletcher and Wolfe, 2016). This finding can be attributed to the family investment model (Zhang, 2021). Child development is not only the result of biological traits and endowments inherited from parents but also the consequence of a parental investment in them. Families with higehr socioeconomic status tends to have richer community resources, allowing parents to establish beneficial learning environments for their children (Boylan et al., 2018). Specifically, they adopt utility-maximizing behaviors and make various forms of investments in their children to enhance their social–emotional competence (Wang et al., 2021). The rich cultural resources, strong human resources and information resources possessed by families with high income, as part of the family cultural capital, are also imprinted in children’s behavioral habits over time. In addition, household disposable income can also lead to intra-household differences in other factors, such as children’s physical health and parents’ emotional expression, which in turn can influence children’s social–emotional competence development (Spinelli et al., 2020).

### 4.2. The direct impact of parental beliefs and interests on social–emotional competence

Interestingly, both parental beliefs and parental interests had a significant effect on children’s social–emotional competence development. This finding reinforces previous findings that without change in structural family characteristics, the stronger the parental beliefs and interests, the better their children’s social–emotional competence develop.

Firstly, in terms of parental beliefs, the stronger the parental beliefs, the better the children’s social–emotional competence. This finding is more consistent with the existing research findings. The Tree-Dimensional theory, which consists of environment, behavior, and individual, suggests that parental beliefs, as an important environmental factor in children’s lives, are effective in transmitting information from the environment to their children and influencing their behavior (Valikhani et al., 2018). In other words, the stronger the parental beliefs and the more importance they place on the development of children’s social–emotional competence, the more information the children receive in the process. The more inclined they will be to observe reciprocity in interpersonal interactions and exhibit more pro-social behaviors that lead to the development of social–emotional competence. For example, Kim concluded that parents’ motivational parental beliefs were significantly and positively related to children’s adaptive functioning, while they were negatively related to children’s externalizing behaviors (Kim et al., 2013). Similar findings were found in a study by Pomerantz et al. They suggest that parental beliefs are a central motivation for parental involvement in children’s education (Pomerantz et al., 2012), parents are more likely to be involved in their children’s social–emotional competence when they believe they are capable of participating in their children’s education and believe it is their role to do so (Cuartas, 2022). This result can be explained by the fact that, on the one hand, parents with high educational beliefs believe strongly in children’s ability to learn, are better able to understand and empathize with children’s behavior. And it is more likely to develop close relationships with children. At the same time, parents with high educational beliefs and interests are more likely than other parents to introduce enriching family learning activities at an earlier point in time, to be more sensitive to children’s demonstrated abilities, and to provide a supportive home learning environment for children (Hadar et al., 2020). Such parents are generally cheerful and open-minded and have a common language for communicating with their children. They understand their children’s needs and are able to provide some inspiration and assistance when necessary, which has a positive effect on children’s social–emotional development.

Secondly, in terms of parental interest, the stronger the parental interest, the better the children’s social–emotional competence. The findings of Roy also validated this finding. If parents have high parental interest, they can help children become aware of their strengths and weaknesses and set learning goals. Such children are generally more likely to exhibit pro-social behaviors and similarly care for their peers, engage in dialog or group negotiation, and have better development of social–emotional skills (Roy and Giraldo-García, 2018). This result can be explained by the fact that parents with strong beliefs and interests enable children to better internalize their educational values. Specifically, parents are the primary source of socialization that children receive in the home. Parents are responsible for teaching children how to manage their emotions, providing them with warm and loving relationships, and ensuring that they live under positive social influences, among other responsibilities (Grusec, 2011). And parental beliefs convey standards of value for their children’s development. At the same time, the socialization process of children is actually the process by which children internalize social value standards. The higher the educational beliefs of parents, the more their children are able to internalize their parents’ role expectations, develop a self-concept as learners, and strengthen their social–emotional competence through interactions with others (Sabato and Eyal, 2022). This phenomenon can be explained by the social exchange theory. This theory suggests that when children accept their parental interest in their social–emotional competence development, they internalize this parental interest as a responsibility and obligation. Thus, they will be more motivated to exhibit the behaviors expected by their parents and to fulfill their parents’ desire for their own social–emotional competence development. Ultimately, this promotes the development of the child’s social–emotional competence (Serna et al., 2022).

### 4.3. Mediating mechanisms of the educational processes

Exploring the mediating role of the educational process is also part of this study.

Firstly, the findings indicate that the educational process mediates the association between structural family characteristics and children’s social–emotional competence. In other words, structural family characteristics can contribute to children’s social–emotional competence not only by influencing family support, but also by influencing home learning activities. This complements previous research findings. That is, the human and social capital of the family does not directly influence children’s social–emotional competence, but rather indirectly influence children’s social–emotional competence through the different educational processes created by parents (Huang et al., 2022).

On the one hand, structural family characteristics contribute to the development of children’s social–emotional competence by influencing family support. Baker’s study also verifies this finding: families are restricted in the material and time resources they can devote to their children when faced with resource constraints (Baker, 2013). In other words, the most direct result of the instability of structural family characteristics is that families are not in a position to meet the need of children’s social–emotional development by providing them with the material resources they need. However, the more stable the structural family characteristics are, the better the parents are at providing good family support for children. According to relational interaction theory, individuals prefer to connect with others who value them and are sensitive to their emotions and needs (Lerner et al., 2015). Parents are more likely to be in this role in families with more durable structural family characteristics. By providing children with appropriate family support, they are able to enable their children to experience a strong sense of autonomy, competence and belonging in close interaction with their parents. In turning, children maintain a positive mental state and enjoy interacting with others. Ultimately, it will foster the development of their social–emotional skills.

On the other hand, structural family characteristics contribute to the development of children’s social–emotional competence by influencing home learning activities. Specifically, the more stable the structural family characteristics, the more materially adequate the family will be. As a result, parents are capable of being more involved in their children’s educational activities and provide positive role models for children, thereby promoting their children’s social–emotional competence. On the contrary, where structural family characteristics cannot be changed, parents and children should be actively required to participate in collaborative family learning activities, thereby stimulating the development of children’s social–emotional abilities. This result can be explained using the Family Stress Model (FSM), which suggests that financial stress is associated with parental emotional distress, which in turn acts to reduce the quality of home learning activities through negative parent–child interactions (Vasilyeva et al., 2018). However, the essence of children’s social–emotional competence is the ‘social construction of relationships’, and the process of developing social–emotional competence is the process of constructing and enhancing relationships (Arace et al., 2021). Parent–child relationships are the earliest and most basic interpersonal relationships for children, and good parent–child interactions promote the formation of intimate parent–child relationships, which enable children to understand themselves and interact with others in a more optimistic manner and provide conditions for their social–emotional development (Wang, 2020).

Interestingly, structural family characteristics as contextual factors influencing children’s development explain, to some extent, who the parents are. However, it is only a crude indicator of what may determine the home learning environment. What parents do is more important than who they are (Šimunović et al., 2018). Therefore, this study does not focus too much on elements that cannot be improved in the short term, such as family socioeconomic status and family demographic variables, but rather on the process of children’s social–emotional competence development. That is, how to improve the quality of the home learning environment and better support the development of children’s social–emotional competencies by enhancing the educational process in the face of limited material resources. These findings are also important for the understanding the intergenerational transmission of poverty (Scorza et al., 2019).

Secondly, the results suggest that the educational process plays a mediating role in the process by which parental beliefs and interests influence preschool children’s social–emotional competence. That is, parental beliefs and interests can influence children’s social–emotional competence not only through family support, but also through family learning activities.

On the one hand, parental beliefs and interests influence the development of children’s social–emotional competence through family support. In other words, parental beliefs and interests are modeled through words, behavior and values in the education process, providing positive role models for children and contributing to the development of their social–emotional competence in a subtle way. This finding is consistent with research related to families from disadvantaged groups. Results from a study of left-behind children in rural China suggest that parental beliefs and interests can negatively affect the development of social–emotional competence in left-behind children (Zhang and Huang, 2022). This may be due to the fact that although parents have strong beliefs and interests, their busy work and fragmented lifestyles result in less time to spend with and educate their children. This prevents them from providing quality family support for children to realize their educational beliefs. Thus presenting a negative impact on children’s social–emotional competence development. This reveals that parents of disadvantaged families should focus on parental beliefs and interests while also promoting the development of children’s social–emotional competencies by improving the quality of family support (Nomaguchi and Milkie, 2020).

On the other hand, parental beliefs and interests influence the development of children’s social–emotional competence through home learning activities. Specifically, the influence of parental beliefs and interests on children is not direct and singular, but requires the transmission of the educational beliefs through interactions with their children, which in turn indirectly influence the development of children’s social–emotional competence (Meloni et al., 2015). This is consistent with previous research findings that parents with higher educational beliefs may influence children’s social–emotional competence development by providing higher quality, warm, and supportive home learning activities (Brackett et al., 2019; Jennings et al., 2020). Parental beliefs are a central motivator for parental involvement in their children’s education. When parents believe that they are capable of participating in their children’s education and that it is their responsibility to do so, they are more likely to engage in frequent parent–child interactions and become more involved in their children’s educational activities (Novianti and Garzia, 2020). And then, parental involvement sends positive signals to the child, who will gain a greater sense of security and importance (Zimmer-Gembeck et al., 2019). In turn, they are more likely to develop high levels of self-esteem and self-efficacy, and are more willing to interact with people other than their parents, promoting the development of children’s social–emotional competence. In addition, the language, behaviors, and values of parents involved in the educational processes provide positive role models for children, which subconsciously promote the development of their social–emotional competence. This result can also be explained by using cognitive control theory (Ma, 2019). Parental beliefs can be understood as their own obligations and responsibilities that need to be fulfilled with respect to a particular social role of the parents. In addition, children also perceive the expectations and beliefs of others (especially ‘significant others’ such as parents) about themselves. Cognitive control theory asserts that individuals need to achieve a unity between these two aspects. When there is a discrepancy between the two, children who are unable to modify their beliefs through the educational process, such as parent–child communication, often experience social and relational pressures that lead to negative mental health states, thus affecting the development of social–emotional competence (Gabrys et al., 2018).

### 4.4. Regulatory mechanisms of gender

This study also examined whether the mediating processes of parental beliefs and interests and structural family characteristics that influence children’s social–emotional competence through the educational processes are moderated by gender. The findings revealed that gender moderates not only the direct path of parental beliefs and interests on children’s social–emotional competence, but also the second half of the mediated path of model a and model b. Specifically:

First, on the direct pathway, parental beliefs and interests had a greater impact on girls’ social–emotional competence compared to boys. This is consistent with previous research that parents have different gender role expectations for their children (Kollmayer et al., 2018). We attempted to apply gender role orientation to explain it (Horne and Johnson, 2018). On the one hand, there are gender differences in parental beliefs and interests influenced by cultural background factors (Bloemen-Bekx et al., 2019). Parents have higher beliefs and interests in social rules and interpersonal relationships for girls and lower boys. It promotes the development of good social relationships in preschool girls and hinders the development of sociality in preschool boys (Tersi and Matsouka, 2020). Ultimately, this affects the development of children’s social–emotional competence. On the other hand, considering children’s own traits, girls are more susceptible to emotional socialization practices due to differences in socialization and expectations (Cui et al., 2020). For example, compared to boys, girls are more sensitive to the emotional messages conveyed by their parents and are more likely to conform to their parental beliefs and gender role requirements. This may lead preschool girls to reinforce and maintain ‘normalized’ pro-social behaviors in order to gain acceptance and affection from parents and others and to gain increased social–emotional development (Wang and Zhang, 2022). However, it has also been suggested that enhanced parental beliefs and attitudes have a greater impact on social–emotional development in boys than in girls (Wang and Zhang, 2012). This differs from the results of the present study and may be explained by the fact that girls develop social–emotional competence earlier than boys in preschool (Behrendt et al., 2020). And this advantage was gradually altered as they grew older (Wong et al., 2022). However, it is not known how the path of change and the final effect. This also provides inspiration for future research.

Second, in the aspect of indirect effects, gender moderated the second half of the pathway of mediating effects between parental beliefs and interests and children’s social–emotional competence, structural family characteristics and children’s social–emotional competence. Indirect facilitation effects of parental beliefs and interests and structural family characteristics on girls’ social–emotional competence were more significant compared to those of boys. That is, in both models a and b, improvements in the quality of the educational processes had a greater impact on girls’ social–emotional competence development compared to boys. This result is in agreement with previous studies (Batool and Lewis, 2020). And it can be attributed to the social construction theory. This theory suggests that there are gender differences in the specific forms of social interaction that can be gained during childhood. And that this pattern of behavior occurs during the continuous interaction between the person and the environment. For children, the interaction process is taken into account in their interaction with their parents (McHarg et al., 2019). On the one hand, parents engage in different types of gender-differentiated activities with their children during their interactions. Parents prefer to engage girls in cooperative, non-cognitive activities that provide more opportunities for social interaction, while boys engage in solitary, educational activities that foster rational and cognitive thinking (Bidzan-Bluma and Lipowska, 2018). This leads to differences in the development of social–emotional skills between boys and girls. On the other hand, girls are often more likely to have a close emotional connection with their parents, which makes parents often involved in education and showing more care and affection (Negraia et al., 2021). In contrast, parents are relatively strict with boys. As a consequence, girls are more likely to be influenced by positive educational processes and show greater social–emotional competence. Boys may consciously control or inhibit emotional expression and empathy in a parenting environment that is relatively devoid of warm interactions, which in turn hinders their social–emotional competence development. However, it has also been suggested that Chinese parents’ education of their children’s social–emotional competence is not influenced by gender compared to other countries (Ren et al., 2016). This differs from the findings of the present study. This may be explained by the fact that the study coincided with the introduction of the one-child policy in China, which limited each family to one child. This initiative made parents attach great importance to their only child. Thus ignoring the gender differences in children (Peng, 2020; Wang and Zhang, 2022).

In summary, the home learning environment is a complex and dynamic system. What’s more, the elements of the home learning environment may change in response to children’s developmental needs as well as the changing nature of the family. The study is groundbreaking in that it explores the synergistic effects of the home learning environment on children’s social–emotional competence development in a more integrated and ecological manner. In particular, the home learning environment is conceptualized to provide reference value for research related to home learning environment. Moreover, the association between the home learning environment and children’s social–emotional competence is typically bidirectional, non-linear, non-additive, and varies according to age. Thus, we can further suppose that there may be a feedback loop effect between the home learning environment and children development. That is, there is a ‘reciprocal’ relationship between children development and the home learning environment, i.e., the home learning environment provided by parents contributes to the development of children’s social–emotional competence. In return, parents are influenced by the children’s development to provide a higher quality home learning environment for children.

### 4.5. Limitations and future directions

The current analysis and findings should also be considered against some limitations regarding the interpretation of our results. Firstly, this study used cross-sectional data for analysis, which is somewhat lacking in verifying the causal relationship. This requires the researcher to further strengthen the longitudinal design for early developmental stages in the future to address such issues more precisely. Secondly, the sample in this study, although representative, is more oriental. It cannot fully describe the overall picture of preschoolers’ home learning environment and social–emotional competence in multiple national contexts. In future studies, the sample could need to be further expanded to investigate children in different countries to improve the generalizability of the findings. Thirdly, this study has only explored the interactions among the dimensions within the home learning environment. However, it has not been explored whether there are more complex interactions between the home learning environment and other environments, especially the interaction with the kindergarten environment, which deserves further research. Therefore, future research should broaden the scope of the field to take a broader view of the dynamic influences between the home learning environment and other environments.

## Data availability statement

The raw data supporting the conclusions of this article will be made available by the authors, without undue reservation.

## Ethics statement

The studies involving human participants were reviewed and approved by Shaanxi Normal University. Written informed consent to participate in this study was provided by the participants’ legal guardian/next of kin.

## Author contributions

SL: conceptualization, formal analysis, and writing original and revised draft. YT: conceptualization, formal analysis, writing original and revised draft, and writing and review. YZ: formal analysis and writing and review. All authors contributed to the article and approved the submitted version.

## Funding

This research was funded by the National Social Science Foundation of China ‘14th Five-Year Plan’ 2022 General Topics in Education. The institutional design and longitudinal study of family education compensation for *de facto* unattended children (BKA220039).

## Conflict of interest

The authors declare that the research was conducted in the absence of any commercial or financial relationships that could be construed as a potential conflict of interest.

## Publisher’s note

All claims expressed in this article are solely those of the authors and do not necessarily represent those of their affiliated organizations, or those of the publisher, the editors and the reviewers. Any product that may be evaluated in this article, or claim that may be made by its manufacturer, is not guaranteed or endorsed by the publisher.
